# Establishment and Analysis of an Individualized EMT-Related Gene Signature for the Prognosis of Breast Cancer in Female Patients

**DOI:** 10.1155/2022/1289445

**Published:** 2022-07-28

**Authors:** Wei Xue, Chenyu Sun, Hui Yuan, Xin Yang, Qiuping Zhang, Yunnuo Liao, Hongwei Guo

**Affiliations:** ^1^Guangxi Key Laboratory for Research and Evaluation of Bioactive Molecules& College of Pharmacy, Guangxi Medical University, Nanning 530021, China; ^2^Key Laboratory of Longevity and Aging-Related Diseases of Chinese Ministry of Education & Center for Translational Medicine, Guangxi Medical University, Nanning 530021, China; ^3^Department of Pharmacy, Ruikang Hospital Affiliated to Guangxi University of Chinese Medicine, Nanning 530011, China; ^4^AMITA Health Saint Joseph Hospital Chicago, 2900 N. Lake Shore Drive, Chicago, 60657 Illinois, USA; ^5^Public Health Center, The First Affiliated Hospital of Xi'an Jiaotong University, Xi'an, 710061, China; ^6^The First Affiliated Hospital of Guangxi Medical University, Guangxi Medical University, Nanning 530021, China

## Abstract

**Background:**

The current high mortality rate of female breast cancer (BC) patients emphasizes the necessity of identifying powerful and reliable prognostic signatures in BC patients. Epithelial-mesenchymal transition (EMT) was reported to be associated with the development of BC. The purpose of this study was to identify prognostic biomarkers that predict overall survival (OS) in female BC patients by integrating data from TCGA database.

**Method:**

We first downloaded the dataset in TCGA and identified gene signatures by overlapping candidate genes. Differential analysis was performed to find differential EMT-related genes. Univariate regression analysis was then performed to identify candidate prognostic variables. We then developed a prognostic model by multivariate analysis to predict OS. Calibration curves, receiver operating characteristics (ROC) curves, *C*-index, and decision curve analysis (DCA) were used to test the veracity of the prognostic model.

**Result:**

In this study, we identified and validated a prognostic model integrating age and six genes (CD44, P3H1, SDC1, COL4A1, TGF*β*1, and SERPINE1). *C*-index values for BC patients were 0.672 (95% CI 0.611–0.732) and 0.692 (95% CI 0.586–0.798) in the training cohort and test set, respectively. The calibration curve and the DCA curve show the good predictive performance of the model.

**Conclusion:**

This study offered a robust predictive model for OS prediction in female BC patients and may provide a more accurate treatment strategy and personalized therapy in the future.

## 1. Introduction

Breast cancer is one of the most prevalent malignancies in women worldwide and the leading cause of most cancer-related deaths, although early-stage BC is considered curable [1, 2]. In 2018, BC was the most commonly diagnosed cancer (24.2% of all cancer cases) and the leading cause of cancer-related deaths (15% of all cancer deaths) in women worldwide. Among these, metastatic BC accounted for more than 90% of BC-related deaths [3]. At present, the main treatment strategies for BC include surgery, chemotherapy, radiotherapy, immunotherapy, and hormonal therapy [4]. Although nanomedicine has been developed this year to target progesterone and estrogen receptors (PR and ER), human epidermal growth factor receptor 2 (HER2), and microRNA (miRNAs) and long chain non-coding RNAs, the incidence of BC remains high, with previous studies suggesting that the number of new cases worldwide will be 2,261,419 women in 2020, and this number is expected to increase to 30.2 million by 2040 [5, 6].

Epithelial-mesenchymal transition (EMT) is widely known to occur during mammalian development, wound healing, and cancer metastasis [7]. In recent years, EMT has received increasing attention for its role in cancer drug resistance [8]. Many studies have shown that EMT is associated with tumorigenesis, invasion, metastasis, and resistance to treatment, especially in BC [9, 10]. Saotome et al. demonstrated that GATA3 truncation mutants affected ductal BC development by altering EMT-related gene expression through partial motif recognition in luminal BC cells [11]. Parthasarathi and his colleagues found that EMT-related genes were associated with dysregulated ion channels in BC-associated tumorigenesis and could potentially be used to determine the prognosis of BC patients. Therefore, in this study, we evaluated the relevance of the EMT genes in female BC patients to explore the mechanisms of EMT in BC [12].

The new 8th edition of a related Union for International Cancer Control (UICC) and American Joint Committee on Cancer (AJCC) publication updates the description of BC staging for tumor lymph node metastasis (TNM) [13]. Yet, it is not sufficient to simply predict the prognosis of BC based on the TNM staging system. Some of the factors that influence BC include age, genes, reproductive factors, estrogen, and lifestyle [14]. Hence, a multifactorial predictive model is essential. Predictive modeling is a more advanced approach as it can be visualized using a nomogram and it can estimate individualized risk based on a more comprehensive set of gene signatures and clinical characteristics. In previous studies, we constructed a clinical prediction model based on the clinical data of metastatic colon cancer patients extracted from the SEER database. The nomogram developed with high prognosis prediction accuracy to evaluate the 1-, 3-, and 5-year survival of metastatic colon cancer patients, which will help clinical decision-making of metastatic colon cancer patients after surgery and individualized treatment [15].

In this study, we identified EMT-related genes with independent prognostic value to establish a prognostic model for predicting the overall survival (OS) at 1-, 3-, and 5-year of female BC patients and generating new insights about BC progression.

## 2. Materials and Methods

### 2.1. Data Collection

We downloaded gene expression data from The Cancer Genome Atlas (TCGA) database (https://cancergenome.nih.gov) of 1109 BC patients and 113 nontumor breast tissues. Clinical data were also acquired, but the clinical data of 12 male BC patients were removed because the study population in this paper was female. EMT-related genes were collected from the Molecular Signature database v7.1 (MSigDB) (http://www.broad.mit.edu/gsea/msigdb/).

### 2.2. Identification of Differentially Expressed EMT-Related Genes

Combining the gene expression data obtained from TCGA database with EMT-related genes by using the “edgeR” package of R software, the expression data of the target genes could be obtained. After that, the “limma” package was utilized to derive differentially expressed EMT-related genes according to False Discovery Rate (FDR) values less than 0.05 and the absolute value of fold change above 1.

### 2.3. Statistical Analysis

#### 2.3.1. Univariate Cox Regression Analysis for Independent Prognostic Factors

The expression matrix of the obtained EMT-related genes was further analyzed by incorporating the matrix with the survival time and survival status. Based on previous studies, age had an impact on the prognosis of female BC patients, so we included age as a study variable [16]. Using the “caret” package in R software (version 4.1.0) to randomly divide the overall cohort into two groups in the ratio of 7 : 3. The subgroup containing 70% of female BC patients was used to construct the prediction model, while the remaining 30% of patients were examined for the accuracy and reliability of the model. Also, the whole cohort was used as the overall internal validation set. The basic values of patients were listed (Table S1).

Univariate Cox regression analysis was used to screen for independent prognostic factors. Factors with a cutoff value of *P* < 0.1 were defined as candidates associated with OS.

#### 2.3.2. Prognostic Nomogram Construction

The genes filtered by univariate Cox regression were then analyzed in the multivariate Cox regression for the risk scoring model. The risk score for each patient can be calculated by the following formula: risk score = Exp(*x*1)∗*β*1 + Exp(*x*2)∗*β*2 + ⋯+Exp(*xn*)∗*βn*, where *n* is the number of selected variables, Exp is the expression level of the variable, and *β* is the regression coefficient of the variable. Then, the nomogram was developed using R software. According to the scores calculated from the nomogram, the patient's OS at 1, 3, and 5 years can be predicted. Subsequently, according to the median risk scores, patients with risk scores greater than the median value were divided into the high-risk group and otherwise into the low-risk group.

#### 2.3.3. Prognostic Nomogram Evaluation and Validation

In order to improve the reliability of the prediction model and thus its clinical application, 30% of the patients and the overall cohort were used as an internal validation cohort to test the validity of the prediction model.

**Figure 1:**
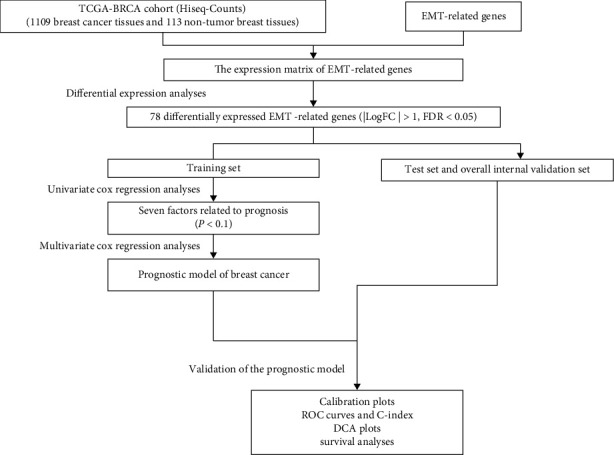
Flow chart of this study.

The discriminative power of the nomogram was calculated using the concordance index (*C*-index). We also measured the area under the curve (AUC) at 1, 3, and 5 years, which was derived from a ROC analysis. The *C*-indexes and AUCs take values ranging from 0.5 to 1.0, where 1.0 represents the perfect ability to correctly distinguish the results from the model and 0.5 represents random chance. The calibration curve of the nomogram was evaluated graphically by plotting the ratio of the predicted probability to the observed ratio of the nomogram. Overlapping with the reference line indicated that the model was perfectly consistent. Finally, decision curve analysis was performed to evaluate the clinical benefits. A flow chart of the study process of this article was presented (Figure 1).

## 3. Results

### 3.1. Identification of Differentially Expressed EMT-Related Genes

**Figure 2:**
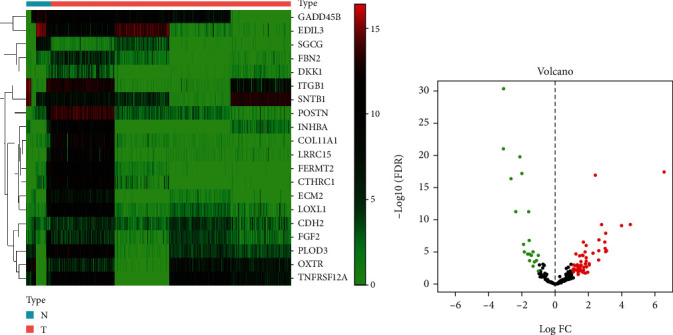
Heat map (a) and volcano map (b) of differentially expressed gene related to EMT.

To describe our study more clearly, we developed a flowchart of the analysis procedure. First, we obtained data from TCGA database for 1109 tumor tissues and 113 nontumor tissues. After taking intersection with EMT-related genes, a matrix of 200 EMT-related genes (Table S2) expression values was acquired. Then, after differential analyses, a total of 78 differentially expressed EMT-related genes were identified. (logFC > 1 or logFC < −1, FDR < 0.05). The results were expressed in heat maps and volcano plots (Figures 2(a) and 2(b)).

### 3.2. Prognostic Nomogram Construction

**Figure 3:**
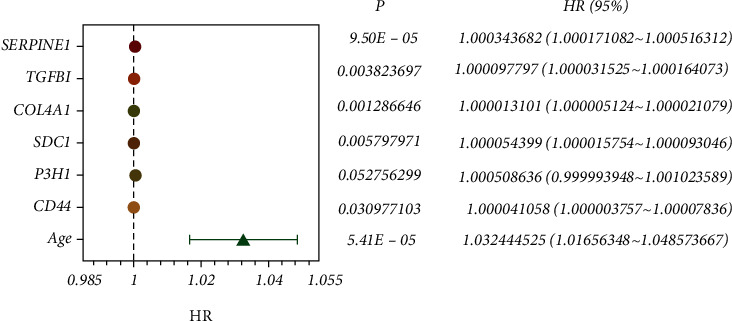
Forest plot analyzed by univariate Cox regression.

Since age is associated with prognosis in female BC patients, we included age and 78 differentially expressed genes in univariate Cox regression to investigate the correlation between the included variables and prognostic value in BC patients and finally identified seven variables significantly associated with OS in BC patients at *P* value < 0.1 (Figure 3). The model was then constructed with age, CD44, P3H1, SDC1, COL4A1, TGF*β*1, and SERPINE1 by multivariate Cox regression: risk score = (0.030655∗age level) + (3.35*E* − 05∗expression level of CD44) + (0.000142∗expression level of P3H1) + (3.76*E* − 05∗expression level of SDC1) + (1.60*E* − 05∗COL4A1 expression level) + (2.04*E* − 05∗TGF*β*1 expression level) + (0.000247∗SERPINE1 expression level) (Table 1).

**Figure 4:**
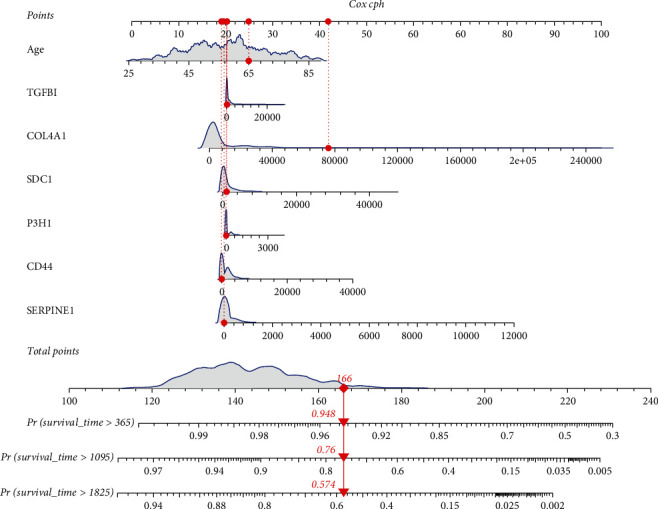
Nomogram for predicting 1-, 3-, and 5-year overall survival (OS) for BC patients in the training cohort.

The nomogram was then constructed and consisted of a total of seven variables (Figure 4), and the total score could be obtained by summing the scores of each variable. The total score can be used to predict the survival rate of individual patients at 1, 3, and 5 years. For example, a BC patient aged 65 years (20 points) with CD44 expression of 0 (20 points), P3H1 expression of 0 (21 points), SDC1 expression of 0 (20 points), COL4A1 expression of 80000 (43 points), TGF*β*1 expression of 0 (21 points), and SERPINE1 expression of 0 (21 points) gets a sum-point of 166, corresponding to predicted 1-, 3-, and 5-year OS of 94.8%, 76.0%, and 57.4%, respectively.

**Figure 5:**
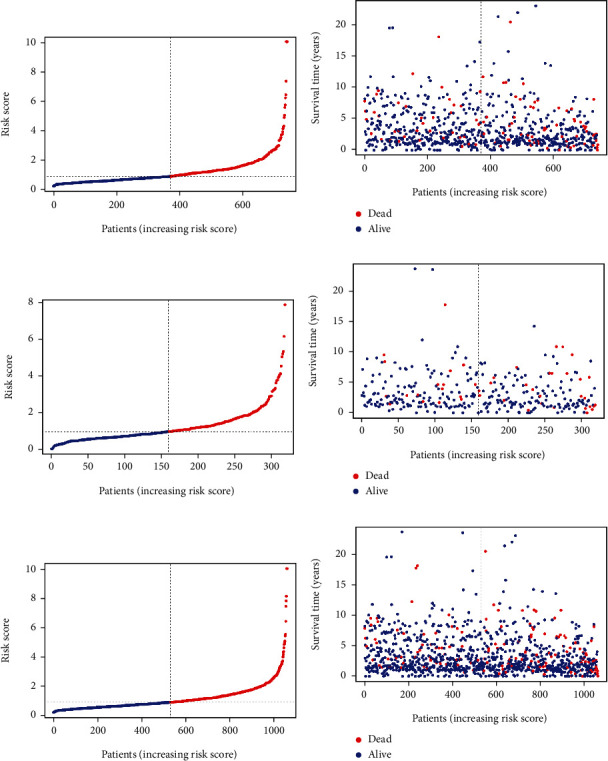
(a, c, e) Distribution of risk score in patients with BC. The black dotted line serves as the dividing line between the high-risk group and the low-risk group. (b, d, f) Diagram of the relationship between risk score and patient survival time. The result of (a, b) is based on training set, the result of (c, d) is based on test set, and the result of (e, f) is based on the overall internal validation set.

**Figure 6:**
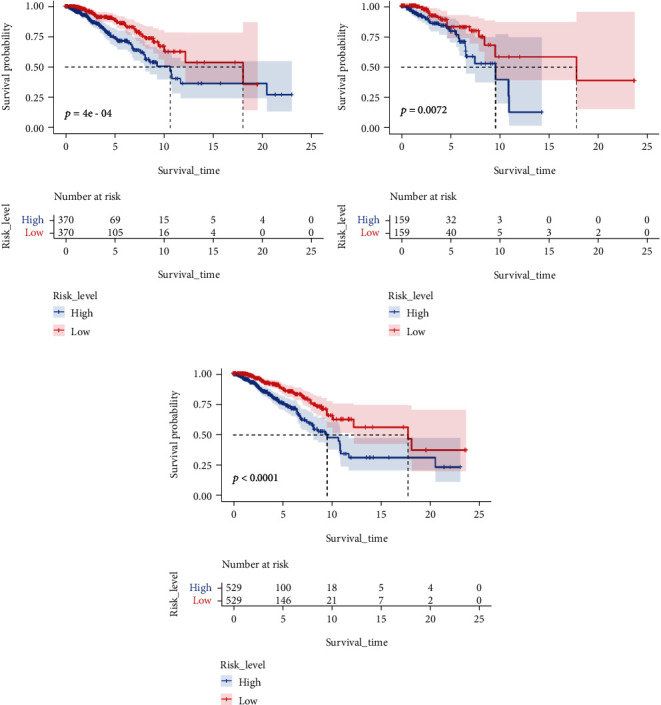
Overall survival (OS) Kaplan-Meier curves for patients in the low- and high-risk groups: (a) training set; (b) test set; (c) overall internal validation set.

Patients in TCGA group were divided into a low-risk group and a high-risk group using the median risk score as the threshold value. Figures 5(a), 5(c), and 5(e) show the distribution of the risk scores of BC patients from high to low in the training set, the internal validation set, and the overall internal validation set. The relationship between risk score and patient survival time in the training set, test set, and overall internal validation set is also shown (Figures 5(b), 5(d), and 5(f)). Patients with high-risk scores tended to have poorer clinical outcomes compared with those with low-risk scores. The survival analyses indicated the high-risk group had worse OS than that of the high-risk group (*P* < 0.05) (Figures 6(a)–6(c)).

### 3.3. Nomogram Calibration and Validation

**Figure 7:**
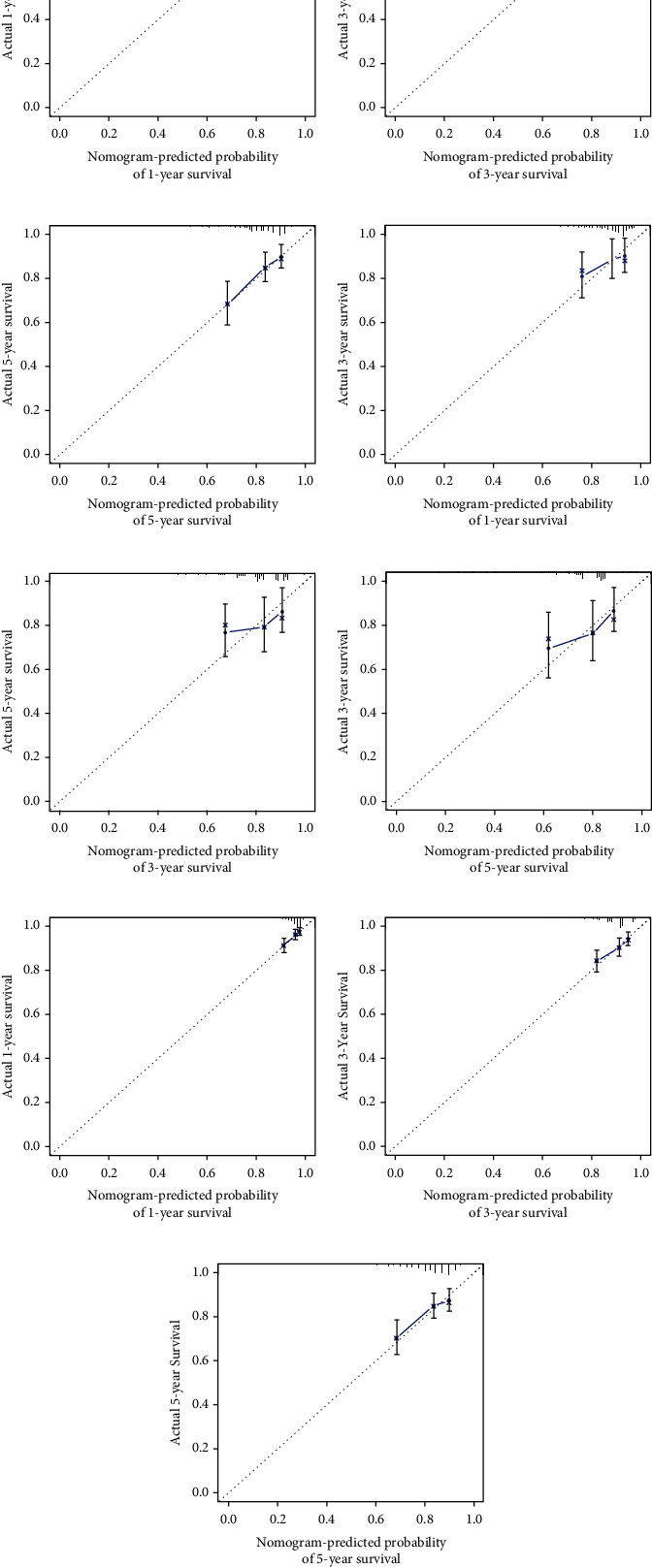
(a–c) Calibration plots to predict 1-, 3-, and 5-year overall survival (OS) in the training set; (d–f) calibration plots to predict 1-, 3-, and 5-year; overall survival (OS) in the test set; (g–i) calibration plots to predict 1-, 3-, and 5-year overall survival (OS) in the overall internal validation set.

The small angle between the survival probability and the actual survival outcome in the calibration curve indicates a strong agreement between them (Figure 7).

**Figure 8:**
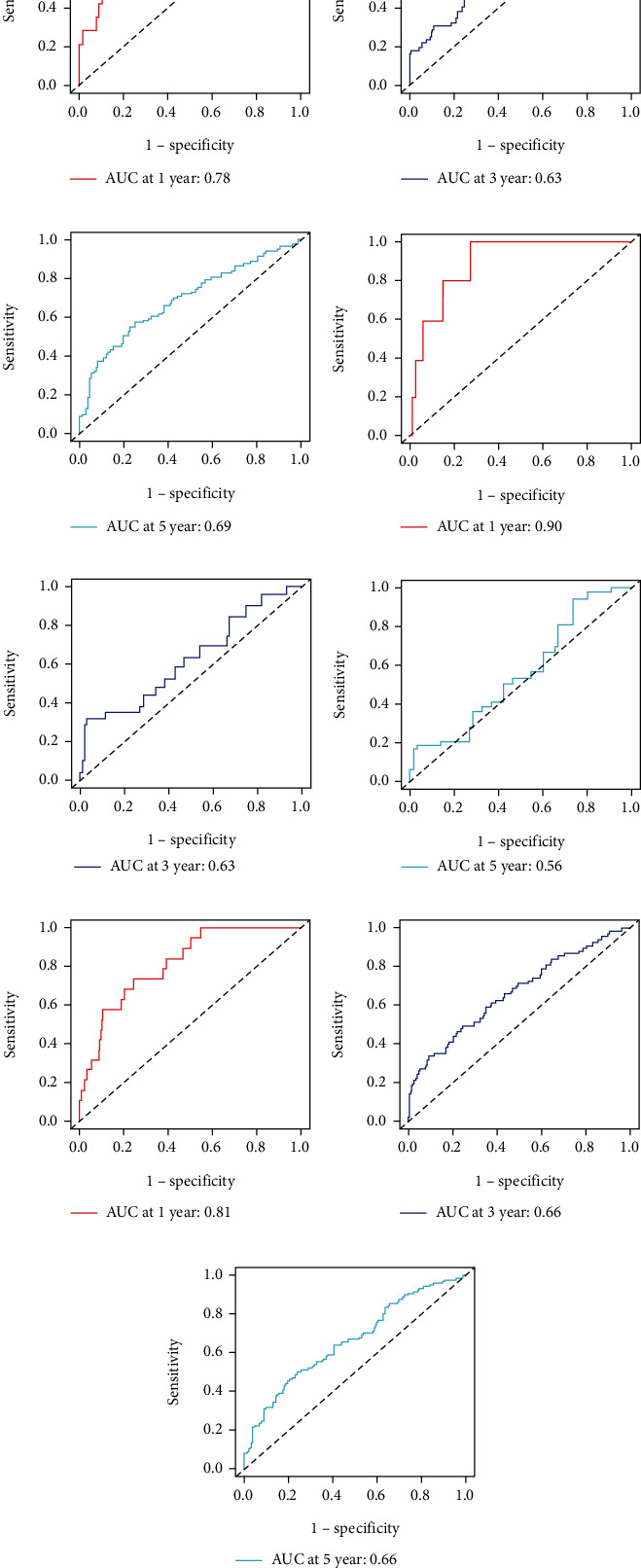
(a–c) ROC curves to predict 1-, 3-, and 5-year overall survival (OS) in the training set; (d–f) ROC curves to predict 1-, 3-, and 5-year; overall survival (OS) in the test set; (g–i) ROC curves to predict 1-, 3-, and 5-year overall survival (OS) in the overall internal validation set.

**Figure 9:**
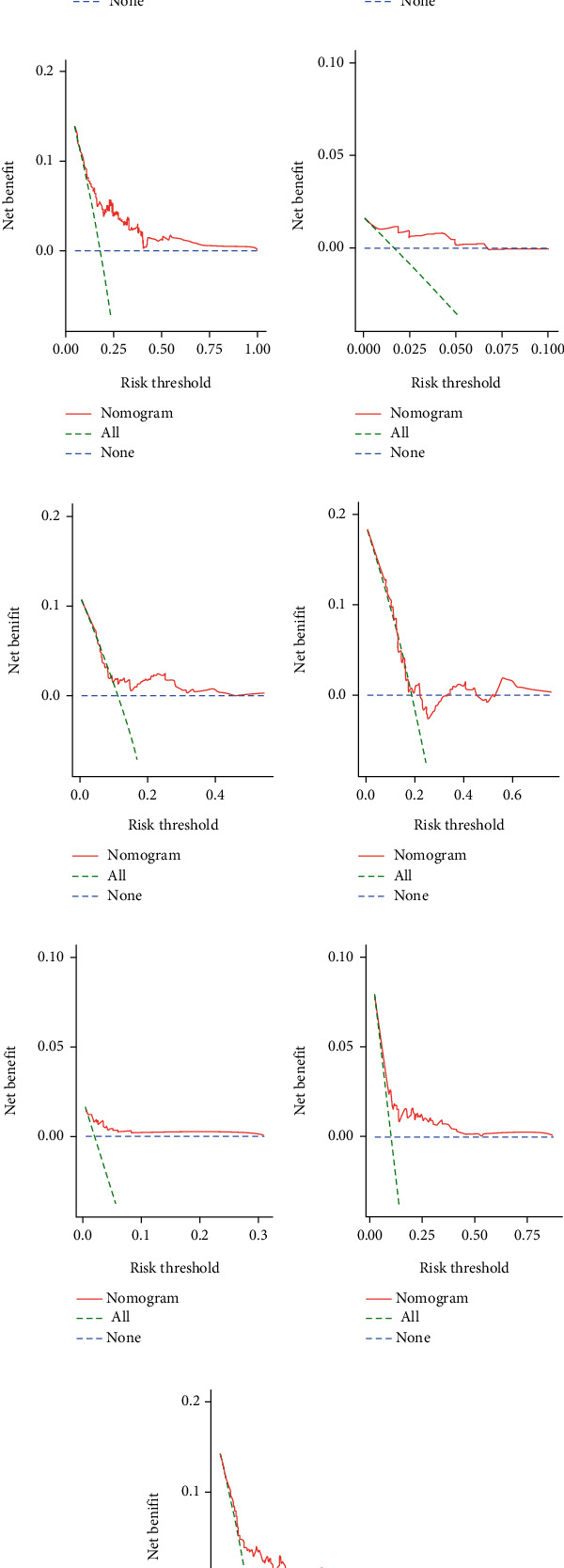
(a–c) DCA analysis predicting 1-, 3-, and 5-year overall survival (OS) in the training set; (d–f) DCA analysis predicting 1-, 3-, and 5-year; overall survival (OS) in the test set; (g–i) DCA analysis predicting 1-, 3-, and 5-year overall survival (OS) in the overall internal validation set.

The *C*-index values for BC patients were 0.672 (95% CI 0.611–0.732), 0.692 (95% CI 0.586–0.798), and 0.679 (95% CI 0.626–0.732) in the training cohort, test set, and overall internal validation set, respectively. The time-dependent ROC curves were used to measure the sensitivity and specificity of the prediction model for predicting OS. Significantly, the AUC values were all greater than 0.63, except for the overall internal validation set with a 5-year predicted survival rate of 0.56, indicating that the model has high survival outcome prediction performance (Figure 8). The DCA curves also revealed better clinical applications for the risk scoring model (Figure 9).

The results based on C-index, ROC curves, calibration curves, and DCA curves indicated that the nomogram in our study demonstrated favorable predictive accuracy for the survival prognosis of female BC patients.

## 4. Discussion

BC is one of the most common cancers in females, with over 1,300,000 new cases and 450,000 deaths worldwide each year [16, 17]. Treatment of BC has advanced considerably, mainly through surgery, neoadjuvant chemotherapy, adjuvant chemotherapy, radiotherapy, systemic therapy, targeted therapy, and so on, with initial conventional surgery no longer being the best option for all patients [1]. However, BC remains one of the leading causes of cancer deaths in women worldwide, largely due to delayed diagnosis and unsuccessful treatment strategies [18]. Therefore, it is crucial to diagnose BC at an early stage and propose a personalized treatment plan based on the characteristics of the women patient's condition to predict their prognosis.

The TNM staging system is still the most widely used prognosis method to predict the survival of patients with BC. Although the American Joint Committee on Cancer (AJCC) updated BC staging in 2016 to include T, N, M, tumor grade, and expression of estrogen and progesterone receptors and HER2 [19], the current TNM staging system still has its undeniable deficiencies. For instance, it does not take into account other pathophysiological characteristics of the patient that have an impact on the prognosis of the tumor: age, gender, exercise, and overweight [20–22]. In addition, gene signature is an important factor in determining the prognosis of BC patients, as BC is a highly heterogeneous disease with different subtypes with different biological, molecular, and clinical processes. Gene expression profiling can identify genetic features to predict prognosis and guide the use of adjuvant therapy [23]. Among others, EMT genes regulate tumor proliferation, invasion, and metastasis [24, 25]. There are many prognostic models on BC; however, this is the first gene signature constructed by EMT-related genes. Moreover, considering the role of age and gender in the onset and progression of BC, we chose to study the prognosis of patients in women and used age as one of the predictors. Compared to previous studies, this nomogram was more accurate.

EMT is a cellular process in which cells lose their epithelial characteristics and acquire mesenchymal characteristics, such as quiescent adnexal cells gaining the ability to migrate [26]. EMT has been associated with a variety of tumor functions, including tumor initiation, malignant progression, tumor stemness, tumor cell migration, intravascular infiltration, metastasis, and resistance to therapy [9]. Most notably in this context, previous studies have shown that both cancer stem cell-like properties and drug resistance are associated with EMT [27]. Given the close link between oncogenic signaling and EMT blockers, EMT has emerged as a therapeutic target or goal in cancer therapy [28]. The relationship between EMT-related genes and breast cancer is also increasingly being investigated by researchers. The major focus of current studies is the regulatory mechanisms and therapeutic approaches of EMT for breast cancer in metastasis and invasion, mainly including miRNA and signaling pathways such as Wnt, Notch, TNF-*α*, NF-*κ*B, and RTK. Investigators suppress breast cancer by attempting to therapeutically target or inhibit key/auxiliary players in these pathways [8, 29–31]. Most notably, upregulation of programmed death ligand 1 (PD-L1) expression is associated with EMT cell phenotype activation, and the control of the interaction between p53 and EMT master regulators is of importance in breast cancer. These two mechanisms have also been studied in other types of cancer and play a key role in the development and metastasis of cancer [30, 32].

This study was based on TCGA database. Differential analysis was firstly performed to find differential EMT-related genes. Univariate regression analysis was then conducted to identify candidate prognostic variables. We then developed a prognostic model by multivariate analysis to predict OS. Calibration curves, receiver operating characteristics (ROC) curves, *C*-index, and decision curve analysis (DCA) were used to test the veracity of the prognostic model. In addition to the training cohort of 70% BC patients, the remained cohort was treated as the test set. In the end, we derived that patient's age, CD44, P3H1, SDC1, COL4A1, TGF*β*1, and SERPINE1 were independent prognostic factors for overall survival in female BC patients and constructed predictive models. The accuracy of the model has also been verified using various methods.

In accordance with our findings, in stage I and IV BC tumors, excess mortality increased linearly with age [33]. Recent studies have shown that a novel positive feedback loop between IL1*β* and CD44 promoted malignant progression in triple-negative BC (TNBC) and that CD44 was a potential target for inhibiting PD-L1 function in TNBC [34, 35]. Sayyad et al. demonstrated the role of Sdc1 in promoting brain metastasis in BC [36]. Several studies have demonstrated that COL4A1 expression could be used as a biomarker for superior prognosis in BC patients receiving neoadjuvant chemotherapy [37], while epigallocatechin-3-gallate (EGCG) exerted antitumor effects by restoring nine key genes, including COL4A1, in myeloid-derived suppressor cells (MDSCs) [37]. TGF*β*1-activated cancer-associated fibroblasts (CAFs) promote BC growth and metastasis in part through autophagy [38]. The evolutionary branch E member 1 (SERPINE1) is a molecule involved in a variety of human malignancies. Zhang et al. showed that SERPINE1 served as an oncogene for PTX resistance in BC, and Xu et al. identified a functional pathway linking miR-1185-2-3p, GOLPH3L, and SERPINE1, which played an essential role in glucose metabolism in BC. Both of their studies revealed that it may serve as a possible target for the treatment of BC [39, 40]. No studies have yet explored the mechanisms by which P3H1 affects BC development, progression, and metastasis, but an algorithm-based meta-analysis of genome-wide and proteomic data identified P3H1 as a potential biomarker for CRC. Our study indicates a direction of research for subsequent basic studies [41].

In this endeavor, some limitations need to be acknowledged. To begin with, the population races in TCGA database are primarily limited to whites and blacks, and extrapolation of findings to other racial groups needs to be validated. Second, a robust nomogram should be externally validated across cohorts; therefore, our nomogram needs to be further validated in multicenter clinical trials and prospective studies. Finally, some of the genes identified in this paper are relatively rarely reported in the academic literature. Therefore, more evidence including sample collection with complete experimental and clinical information should be performed for future validation is needed to elucidate the intrinsic association between age and six-gene signature and prognosis of BC patients.

However, our study also has some advantages. To our knowledge, this is the first study to additionally combine age as a prognostic variable with EMT-related genes to predict the prognosis of BC patients. Prognostic models may predict patient prognosis more accurately than conventional indicators.

In conclusion, we have developed and validated a relatively effective predictive model to predict the survival outcome of female BC patients at 1, 3, and 5 years. The accuracy and reliability of the prognostic model have also been verified. The results of our research need to be further validated in subsequent clinical practice.

## Figures and Tables

**Table 1:** Genes contained in the prognostic model of breast cancer.

Factors	coef	HR	HR_95L	HR_95U	*P*
Age	0.030655	1.031129	1.015051	1.047463	0.000132
CD44	3.35*E* − 05	1.000033	0.999994	1.000073	0.098155
P3H1	0.000142	1.000142	0.999283	1.001002	0.74605
SDC1	3.76*E* − 05	1.000038	0.999966	1.000109	0.304272
COL4A1	1.60*E* − 05	1.000016	1.000008	1.000024	0.000149
TGFBI	2.04*E* − 05	1.00002	0.999884	1.000156	0.768605
SERPINE1	0.000247	1.000247	0.999911	1.000583	0.149765

## Data Availability

The research article data used to support the findings of this study are included within the article.

## Supplementary Materials

Supplementary Materials Supplementary Table S1: basic values of patients. Supplementary Table S2: EMT-related gene list.
