# Supplementary material for: Establishment and Analysis of an Individualized EMT-Related Gene Signature for the Prognosis of Breast Cancer in Female Patients
Source: Dis Markers. 2022 Jul 28;2022:1289445. doi: 10.1155/2022/1289445 (PMC9352481; doi:10.1155/2022/1289445)
Supplement: Supplementary Materials — Supplementary Table S1: basic values of patients. Supplementary Table S2: EMT-related gene list. [file 1289445.f1.zip › Supplementary materials Table S2.docx]

EMT-related gene list

ABI3BP

ACTA2

ADAM12

ANPEP

APLP1

AREG

BASP1

BDNF

BGN

BMP1

CADM1

CALD1

CALU

CAP2

CAPG

CCN1

CCN2

CD44

CD59

CDH11

CDH2

CDH6

COL11A1

COL12A1

COL16A1

COL1A1

COL1A2

COL3A1

COL4A1

COL4A2

COL5A1

COL5A2

COL5A3

COL6A2

COL6A3

COL7A1

COL8A2

COLGALT1

COMP

COPA

CRLF1

CTHRC1

CXCL1

CXCL12

CXCL6

CXCL8

DAB2

DCN

DKK1

DPYSL3

DST

ECM1

ECM2

EDIL3

EFEMP2

ELN

EMP3

ENO2

FAP

FAS

FBLN1

FBLN2

FBLN5

FBN1

FBN2

FERMT2

FGF2

FLNA

FMOD

FN1

FOXC2

FSTL1

FSTL3

FUCA1

FZD8

GADD45A

GADD45B

GAS1

GEM

GJA1

GLIPR1

GPC1

GPX7

GREM1

HTRA1

ID2

IGFBP2

IGFBP3

IGFBP4

IL15

IL32

IL6

INHBA

ITGA2

ITGA5

ITGAV

ITGB1

ITGB3

ITGB5

JUN

LAMA1

LAMA2

LAMA3

LAMC1

LAMC2

LGALS1

LOX

LOXL1

LOXL2

LRP1

LRRC15

LUM

MAGEE1

MATN2

MATN3

MCM7

MEST

MFAP5

MGP

MMP1

MMP14

MMP2

MMP3

MSX1

MXRA5

MYL9

MYLK

NID2

NNMT

NOTCH2

NT5E

NTM

OXTR

P3H1

PCOLCE

PCOLCE2

PDGFRB

PDLIM4

PFN2

PLAUR

PLOD1

PLOD2

PLOD3

PMEPA1

PMP22

POSTN

PPIB

PRRX1

PRSS2

PTHLH

PTX3

PVR

QSOX1

RGS4

RHOB

SAT1

SCG2

SDC1

SDC4

SERPINE1

SERPINE2

SERPINH1

SFRP1

SFRP4

SGCB

SGCD

SGCG

SLC6A8

SLIT2

SLIT3

SNAI2

SNTB1

SPARC

SPOCK1

SPP1

TAGLN

TFPI2

TGFB1

TGFBI

TGFBR3

TGM2

THBS1

THBS2

THY1

TIMP1

TIMP3

TNC

TNFAIP3

TNFRSF11B

TNFRSF12A

TPM1

TPM2

TPM4

VCAM1

VCAN

VEGFA

VEGFC

VIM

WIPF1

WNT5A
